# An inoculum-dependent culturing strategy (IDC) for the cultivation of environmental microbiomes and the isolation of novel endophytic Actinobacteria

**DOI:** 10.1038/s41429-019-0226-4

**Published:** 2019-08-29

**Authors:** Mohamed S. Sarhan, Elhussein F. Mourad, Rahma A. Nemr, Mohamed R. Abdelfadeel, Hassan-Sibroe A. Daanaa, Hanan H. Youssef, Hanan A. Goda, Mervat A. Hamza, Mohamed Fayez, Bettina Eichler-Löbermann, Silke Ruppel, Nabil A. Hegazi

**Affiliations:** 1 Department of Microbiology, Faculty of Agriculture, Cairo University, Giza, Egypt; 2 Faculty of Agricultural and Environmental Sciences, Rostock University, Rostock, Germany; 3 Department of Genetics, School of Life Science, the Graduate University for Advanced Studies (SOKENDAI), 1111 Yata, Mishima, Shizuoka 411-8540 Japan; 4 Leibniz Institute of Vegetable and Ornamental Crops (IGZ), Großbeeren, Germany

**Keywords:** Microbiology techniques, Bacteriology, Microbial ecology

## Abstract

The recent introduction of plant-only-based culture media enabled cultivation of not-yet-cultured bacteria that exceed 90% of the plant microbiota communities. Here, we further prove the competence and challenge of such culture media, and further introduce “the inoculum-dependent culturing strategy, IDC”. The strategy depends on direct inoculating plant serial dilutions onto plain water agar plates, allowing bacteria to grow only on the expense of natural nutrients contained in the administered inoculum. Developed colonies are successively transferred/subcultured onto plant-only-based culture media, which contains natural nutrients very much alike to those found in the prepared plant inocula. Because of its simplicity, the method is recommended as a powerful tool in screening programs that require microbial isolation from a large number of diverse plants. Here, the method comfortably and successfully recovered several isolates of endophytic Actinobacteria represented by the six genera of *Curtobacterium* spp., *Plantibacter* spp., *Agreia* spp., *Herbiconiux* spp., *Rhodococcus* spp., and *Nocardioides* spp. Furthermore, two of the isolates are most likely novel species belonging to *Agreia* spp. and *Herbiconiux* spp.

Not-yet-cultured populations represent diverse groups of microbes that account for more than 90% of a given ecosystem that are still under shadow [1]. To gain insights into their unknown functions and exploit their potentials, different approaches were recently introduced to culture such not-yet-cultured bacteria [2, 3]. In this respect, the plant-only-based culture media were presented as natural culture media to replace myriad formulas of synthetic culture media, and strongly recommended to increase the cultivability of the plant microbiota [4–10]. To alleviate the stress of disproportionate nutrients, present in common culture media, we aimed at culturing maize and sunflower microbiota on the natural nutrients present in the plant inoculum itself, compared to highly diluted plant-only-based culture media and standard R2A. The rationale of the study is to allow the existing plant microbiota to grow on nutrient-deficient water agar (WA) depending on the proportionate nutrients present in the administered inoculum itself. Expressly, the plant inoculum is used as a dual source of enclosed microbes and inherent nutrients.

To test this hypothesis, appropriate serial dilutions of surface-sterilized roots (rhizosphere) and shoots (phyllosphere) of maize plants (*Zea mays* L.) were surface-inoculated on plain WA, principally used to examine the growth of colony-forming units (CFUs) of plant endophytes depending on the nutrients contained in the inoculum itself. For comparisons, i.e., bench marking, other sets of agar plates were prepared as well from: (a) 1/200 diluted autoclave-sterilized maize juice (AJ, v/v), (b) 1/200 diluted filter-sterilized maize juice (FJ, v/v, filtration through 0.2 µm Sartorious membrane filters), and (c) 1/100 diluted (v/v) R2A culture medium. The plant juices were prepared by juicing carefully washed shoots of full-grown plants using a sugarcane juicer. By incubation at 25 °C, counts of CFUs of the rhizosphere, log 8.13–8.75 g^−1^, markedly exceeded those reported for phyllosphere, log 5.23–5.51 g^−1^ (Fig. 1a). In general, CFU counts were lowest at 4 days of incubation, and prolonging the incubation period up to 32 days resulted in slight, but not always significant, increases in CFU counts for either spheres. The extension of incubation time significantly stimulated the development of microcolonies (i.e., µCFU, <1 mm dia.). Notably, the development of such microcolonies was very much reported for the rhizosphere compartment by the end of the incubation period (32 days), representing >20% out of total CFU counts (Fig. S1). Such phenomenon of microcolonies development was brought into focus while introducing novel approaches for increasing culturability of environmental microbiomes [2, 11, 12]. Indeed, the development of microcolonies was significantly enhanced by new methodologies for culturing the uncultured bacterial populations, e.g., the use of overlay agar technique for plating [13], the diffusion-chamber-based techniques, encapsulation of cells in gel microdroplets under low nutrients flux conditions, and soil slurry membrane system that combines a polycarbonate membrane as a growth support and soil extract as substrate [14]. Majority of microcolonies developed by such techniques were isolated in pure cultures and identified as not-yet-cultured/novel species bacteria [14]. The recurrent development of such microcolonies was experienced in the present study, depending on the combined effect of prolonged incubation time and the proportionate nutrient concentrations present either in WA‚ receiving plant nutrients contained in the plant inoculum itself, or the extensively diluted plant juice and R2A agar. In other words, the limited nutrients, present in such extensively diluted culture media, do not satisfy the growth of fast-growing bacteria, but allow the recovery of fastidious ones developed in form of microcolonies.

**Fig. 1:**
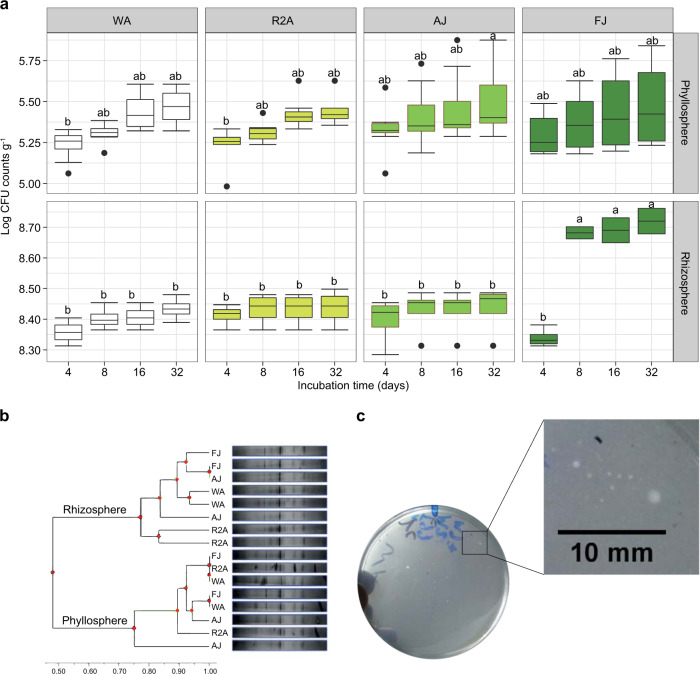
Culture-dependent bacterial community analysis of maize rhizosphere and phyllosphere. **a** Log CFU counts developed on the tested culture media over time. Statistically significant differences are designated by different letters based on Tukey’s Honestly Significant Differences (HSD, *P* ≤ 0.05, *n* = 4). **b** DGGE analysis of 16S rRNA gene profiling of culturable bacterial community recovered on the tested culture media after 32 days of incubation, clustered with UPGMA method based on Euclidean distances (totallab.com/home/cliqs). **c** Morphologies of colonies grown on WA together with zoom in section of macro- and micro-colonies. Scaling was carried out using ImageJ software (imagej.nih.gov/ij). WA water agar, R2A 1/100 diluted (v/v) R2A, AJ autoclaved 1/200 diluted (v/v) maize juice agar, and FJ filtered 1/200 diluted (v/v) maize juice agar culture media

Although the limitations of PCR-DGGE of the 16S rRNA gene, the method is nonetheless in practice for studying microbial diversity [15]. Consequently, we used culture-dependent PCR-DGGE of the 16S rRNA gene to analyze the community compositions of the culturable endophytes of maize rhizosphere and phyllosphere [7, 16]. PCR-DGGE analysis of CFUs harvest clearly differentiated the rhizosphere populations from those inhabiting the phyllosphere at similarity level of 48% (Fig. 1b). This might be attributed to the differential composition of the nutrient pools of either plant compartments. No explicit separations were reported as due to the culture media effect. Hence, it is suggested that by using highly diluted culture media, the inoculum has stronger impact in shaping culturable community composition than the culture medium by itself. This is in consistency with the findings of Hegazi et al. [6]. They reported gradual separations of DGGE profiles of culturable population of Lucerne rhizobacteria according to nutrient concentrations and origin; at the very low concentrations, i.e., 0.25–1.00 g l^−1^ of plant powder, no further separations were observed. Such phenomenon, of source inoculum effect, was defined as the influence of the inoculum on the growth of its pertinent load of bacterial cells.

To confirm such phenomenon, another experiment was carried out with maize phyllosphere. This is primarily to test the pretreatment of inocula suspensions to eliminate as much as possible their contents of nutrients’ traces. This pretreatment included low-speed centrifugation for the removal of coarse plant debris and high-speed centrifugation for collecting pellets of bacterial cells, together with successive washings (Supplementary methods). Results indicated highly significant differences (*P* = 2.24 × 10^−6^) attributed only to the inoculum pretreatment (Fig. S2). The significant decreases in counts of CFUs developed on WA not AJ is a clear indication on the affinity between the nutrients and the bacterial load of the inocula.

In view of the previous results obtained, we further analyzed the phyllosphere bacteria of sunflower plant (*Helianthus annuus* L.) using the IDC strategy to confirm the microcolonies phenomenon and specifically analyze their taxonomic affiliation. In general, counts of CFUs developed on culture media of AJ and FJ of sunflower, diluted R2A, and plain WA were in the range of log 3.9–log 4.6 g^−1^. With prolonged incubation (9 days), one-way ANOVA displayed no significant differences, and counts of CFUs developed on the plain WA were very much comparable with those developed on the conventional culture media based on plant juices (AJ and FJ) or chemically synthetic nutrients (R2A) (Table 1). Again, the pervasive occurrence of microcolonies was reported on both WA and FJ. Randomly, we picked representatives of those microcolonies (µCFUs) for further subculturing and taxonomic identification using 16S rRNA gene sequencing. Isolates derived from WA plates failed to further subculturing on plain WA. Interestingly however, all of the isolates, including those recovered from WA, successfully maintained culturability on FJ semi-solid and agar culture media for up to 4–6 subsequent generations. This is a further proof that IDC is a workable culturing strategy of potential application as a culturomic tool in future. As to the microcolonies, some retained their status (<1 mm dia.), while others developed into macrocolonies (>1 mm dia.), with varying chromogenic phenotypes. This is contrary to what have been earlier reported by previous investigations, where microcolonies were irreproducible, i.e., they were not able to grow when subcultured on the same original culture media of isolation [8, 12, 17]. It appeared that microcolonies require further and careful domestication/passage process to sustainably grow in standard petri dishes [18]. In our case, microcolonies were able not only to just survive after subculturing, but also proliferate and turn into macrocolonies when further subcultured on the related plant-only juice culture media (FJ).

**Table 1:** Log CFU counts of phyllosphere bacteria of sunflower plants developed on all tested culture media over incubation time

Culture media	Incubation time (days)	
	4 days	9 days
Plain water agar (WA)	3.94 ± 0.23^d*^	4.20 ± 0.26^bcd^
Standard R2A	4.45 ± 0.20^ab^	4.52 ± 0.18^ab^
Autoclave-sterilized sunflower juice (AJ)	4.05 ± 0.32^cd^	4.39 ± 0.29^abc^
Filter-sterilized sunflower juice (FJ)	4.48 ± 0.20^ab^	4.60 ± 0.15^a^

^*^Statistically significant differences are designated by different letters based on Tukey’s Honestly Significant Differences (HSD, *P* ≤ 0.05, *n* = 4)

The 16S rRNA gene sequences of secured isolates were deposited in the GenBank under the accession numbers MK100479-MK100506, and compared with the databases of GenBank (ncbi.nlm.nih.gov) and EZBioCloud (ezbiocloud.net). The analysis revealed the domination of endophytic Actinobacteria species, with single incidences of each of the phyla Proteobacteria (*Methylobacterium mesophilicum*) and Firmicutes (*Bacillus aryabhattai*). The 26 isolates of the phylum Actinobacteria represented the genera *Curtobacterium* spp. (18 isolates), *Plantibacter* spp. (1 isolate), *Agreia* spp. (1 isolate), and *Herbiconiux* spp. (1 isolate) of the family Microbacteriaceae, as well as the genus *Rhodococcus* spp. (2 isolates) of the family Nocardiaceae, and the genus *Nocardioides* spp. (2 isolates) of the family Nocardioidaceae (Fig. 2). Interestingly, the isolates F19 and F20 are most likely to represent novel species—their highest matching scores were <98.7%. This was confirmed as Maximum likelihood phylogeny, with bootstrapping of 1000 replicates, displayed significant separation of such two isolates away from all of the deposited members of the genera *Herbiconiux* and *Agreia* (Fig. 2).

**Fig. 2:**
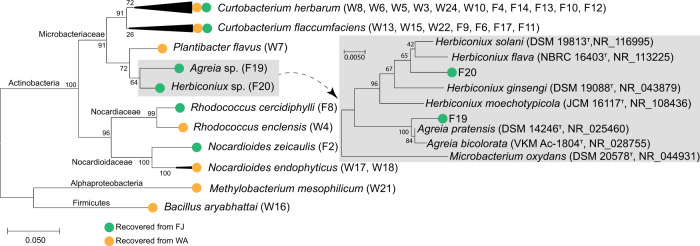
Maximum likelihood (ML) phylogenetic tree of the bacterial isolates recovered from WA (15 isolates) and FJ (13 isolates) culture media, based on 16S rRNA gene sequences. The subtree includes the isolates F19 and F20 with all reported species of the genera *Agreia* and *Herbiconiux*, including *Microbacterium oxydans* as an outgroup. Bootstrapping was performed for each tree with 1000 replicates; the percentage of trees in which the associated taxa clustered together is shown next to the branches. Phylogenetic analyses were conducted in MEGA X. WA water agar, R2A 1/100 diluted (v/v) R2A, AJ autoclaved 1/200 diluted (v/v) sunflower juice agar, and FJ filtered 1/200 diluted (v/v) sunflower juice agar culture media

Our results highlight the endophytic nature of Actinobacteria, as well as their wide occurrence in plant tissues, being widely known as a potential bio-repertoire of natural products. To facilitate their isolation and cultivation, tens of culture media were developed and enabled isolation of wide range of Actinobacteria, as described in literature (Table S1). They all contain one or more of diversified carbon and nitrogen sources, as well as growth factors, e.g., soluble starch, lignin, chitin, cellulose, glycerol, asparagine, casein, yeast extract, soil extract, humic acid, and consortia of vitamins and amino acids [19, 20]. Culture media derived from plant materials (e.g., oatmeal medium, YIM21) are also recommended [19]. It appeared that a prerequisite of culturing fastidious Actinobacteria is to suppress fast-growing microbes and eliminate competition of Gram-positive and Gram-negative bacteria, as well as fungi. This is achieved through use of complex C and N sources, salt concentrations, pH levels, and appropriate antibiotics and chemicals. This is imperative to keep mining the rich store of antibiotic, active compounds, and secondary metabolites of newly isolated Actinobacteria [21, 22].

As the WA does not support growth of most prokaryotes per se [23], the reported ability of WA to recover CFU counts implies that the applied inocula contained both microbes (biomass) and nutrients (plant juice). In theory, the constitutive effect of the source inoculum on CFU development is considered of an intrinsic nature. When using conventional rich culture media, such effect is suppressed, i.e., being recessive. Employing the host plant materials (e.g., juices, concentrates and powders) in the preparation of plant-based culture media, as in Mourad et al. and Hegazi et al. [5, 6], the effect appears as synergic. In case of using IDC or highly diluted culture media, the effect appears as dominant. This persuades us to reconsider previous reports on the use of diluted culture media and the resulted increases in culturability [6, 24]. We may extrapolate that such increases are most likely considered as an inoculum effect. This supports the growing interest in strategies of increasing cultivability of in situ bacterial communities by boosting the recovery of microcolonies via dilution of nutrients present in the recommended standard culture media and/or adjusting in vitro atmosphere of the surroundings [2, 17].

In conclusion, our results encourage the use of ultra-diluted plant-only-based culture media, contained in the plant inoculum itself (IDC), for in vitro cultivation of the plant microbiota. The method guarantees providing real-time nutrients of the tested homologous host plants with their unique diversity, complexity, and concentrations. This strategy is a simple, practical, and convenient approach to mine for the hidden and novel members of the plant microbiota, particularly those of biotechnological potential like Actinobacteria. It is a promising culturomic tool, and highly recommended for future screening programs that require isolation of large number of isolates from diverse plants, as well as multiple analysis of other environmental microbiomes.

## Supplementary information

Supplementary material - Clean copy
