# Supplementary material for: An inoculum-dependent culturing strategy (IDC) for the cultivation of environmental microbiomes and the isolation of novel endophytic Actinobacteria
Source: J Antibiot (Tokyo). 2019 Aug 29;73(1):66–71. doi: 10.1038/s41429-019-0226-4 (PMC8075983; doi:10.1038/s41429-019-0226-4)
Supplement: Supplementary file 1 — Supplementary material - Clean copy [file 41429_2019_226_MOESM1_ESM.doc]

## Supplementary materials

**An inoculum-dependent culturing strategy (IDC) for the cultivation of environmental microbiomes and the isolation of novel endophytic Actinobacteria**

**Supplementary methods**

**Host plant sampling**

Representative samples of the whole root system and the leaves of full-grown maize plant (*Zea mays*) were obtained from the experimental fields of the Faculty of Agriculture, Cairo University, Giza, Egypt. The sunflower plants (*Helianthus annuus*) were obtained from the experimental fields of the Leibniz Institute of Vegetables and Ornamental Crops (IGZ), Großbeeren, Germany. Microbiological analyses were conducted within 2 hours of sampling.

**Preparation of culture media**

**Plant-culture media, based on filter- and autoclave-sterilized juices:** The maize juice of vegetative shoots and leaves was extracted by small electrical sugarcane juicer, while sunflower leaves were crushed in Waring blender. Crude juices were then pre-filtered through Whatman No. 1 filter papers and stored at -20°C until usage. Two formulations of plant-only-based culture media were prepared from sterilized juices, either autoclaved (for 20 min at 121°C) or filtered (through Sartorius membrane filters of 0.2 µm pore size). The prepared juices were added to autoclaved water agar to a final concentration of 1:200 (v/v) to prepare plant-only-based culture media: filter-sterilized juice **(FJ)** and autoclave-sterilized juice **(AJ)**. The measured pH of both culture media was 7.0 ± 0.1.

**Water agar culture medium (WA):** Bacteriological Agar (residue on ignition (Ash) < 6.5%, Sigma-Aldrich A5306, MO, USA) was added to distilled water (1.2%, w/v), then autoclaved for 20 min at 121°C.

**Reasoner’s 2A agar culture medium (R2A):** The R2A culture medium was prepared according to Reasoner and Geldreich [1], then diluted with distilled water 1:100 (v/v).

All tested culture media were tested for sterility before use.

**Recovery of endophytic bacterial populations:**

Microbiological analyses were carried out for both the endorhizosphere and endophyllosphere communities of maize plants and the phyllosphere of sunflower. Initial suspensions of tested plant spheres were prepared by adding 5 g of the respective plant samples to 45 mL of the sterilized ½ strength basal salts of CCM [2], and carefully blended with mortars and pestles. Further serial dilutions were prepared, and aliquots of 100 μL of suitable dilutions were surface-inoculated on agar plates prepared from tested plant-only-based and R2A culture media. Incubation took place at 25°C for up to 32 days for maize and at 28°C for up to 9 days for sunflower.

**Elimination of traces of nutrients present in plant inocula**

In an attempt to remove as much as possible of plant nutrients in the original maize phyllosphere suspension, one mL of the suspension was transferred into a 1.5 mL microcentrifuge tube and low-speed centrifuged (at 3,000 rpm for 30 sec) for the removal of coarse plant debris. The supernatant was transferred into another microcentrifuge tube and re-centrifuged at 10,000 rpm for 10 min. The resultant supernatant was discarded and the pellet was re-suspended in ½ strength basal salts of CCM; the last two steps were repeated thrice [3]. Then, further serial dilutions were prepared, and aliquots of 100 μL of suitable dilutions were surface inoculated on agar plates of AJ and WA culture media. Incubation took place at 25°C for up to 32 days. Developed colonies were differentiated to macro-colonies (MCFU, ≥ 1 mm in diameter) and micro-colonies (µCFU, < 1mm in diameter).

**DGGE analysis of culture-dependent bacterial communities**

Maize endophytic CFUs developed on agar plates (>30–300 CFUs plate^–1^), in 2 replicates representing the various culture media, were harvested using 4 mL of 0.05 M NaCl solution. CFUs harvest suspensions were centrifuged for 10 min at 13000 rpm. DNA extraction was carried out from the resulting pellets using i-genomic DNA Extraction Mini Kit (iNtRON Biotchnology Inc., Kyungki-Do, Korea). DNA concentration was measured by BioDrop µLITE Spectrophotometer (BioDrop Inc., Warwickshire, UK).

Amplification of whole 16S rRNA gene and reamplification of the V3-region were carried out according to Sarhan et al. [4], using the universal primers of Muyzer et al. and Mühling et al. [5, 6].

DGGE was performed according to Sarhan et al. [4], with VS20WAVE-DGGE (Cleaver Scientific, Warwickshire, UK). DGGE fingerprints were analyzed using CLIQS 1D software (TotalLab, Newcastle upon Tyne, UK).

**DNA extraction and 16S rRNA gene sequencing of representative isolates of sunflower phyllosphere**

Surface-inoculated plates were prepared for tested sunflower phyllosphere, representing various tested culture media, of FJ, AJ and R2A. After 9 days of incubation at 28°C, CFUs were carefully monitored. Representative micro-colonies (< 1.0 mm in diameter) particularly developed on water agar (16 colonies) and FJ culture media (16 colonies) were further subcultured on agar plates of plant-juice-based culture media. On FJ semi-solid culture media and agar plates, all of the isolates were successfully subcultured and maintained growth for several passages. They were subjected to DNA extraction and 16S rRNA gene sequencing (Eurofins MWG Operon, Ebersberg, Germany). To obtain the nearest phylogenetic neighbours, 16S rRNA gene sequences were compared with their closest matches using EZBioCloud identification tool ([ezbiocloud.net/identify](https://www.ezbiocloud.net/identify)). The Sequence alignments and the phylogenetic tree were constructed using ClustalW and the Neighbour-Joining method based on the Jukes-Cantor model implemented in MEGA X [7]. The 16S rRNA gene sequences identified in this study have been deposited in the GenBank database under the accession numbers MK100479-MK100506.

**Statistical analysis**

The following R-CRAN packages ([cran.r-project.org](../cran.r-project.org)): “Agricolae” for calculating the Honest Significant Differences (HSD) and “ggplot2” for plotting.


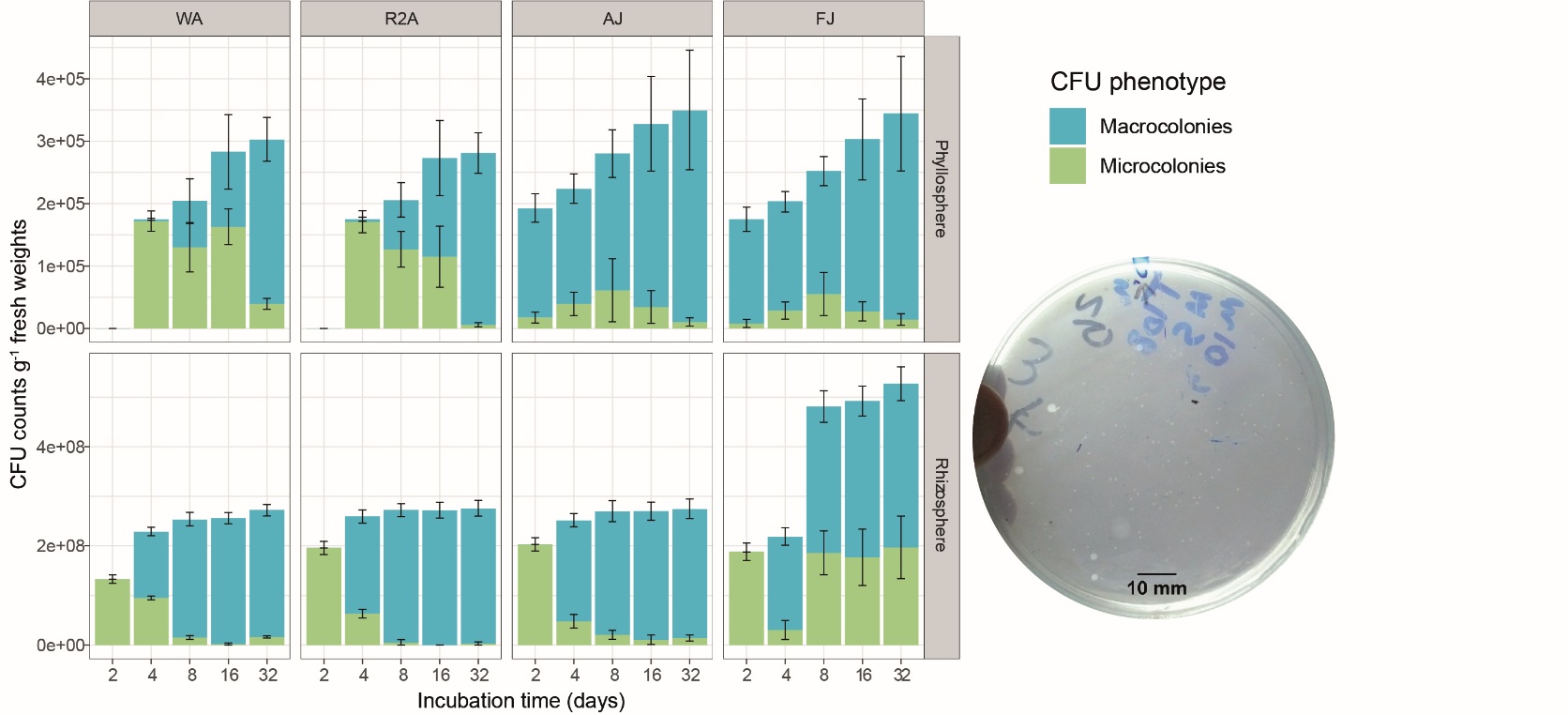
 **Fig. S1** CFU counts of bacterial community analysis of maize rhizosphere and phyllosphere. Bars indicate the proportion of micro- and macro-colonies. Error bar indicates the standard error (n = 4). Inserted is a plate showing the development of macro- and micro-colonies on water agar (WA).


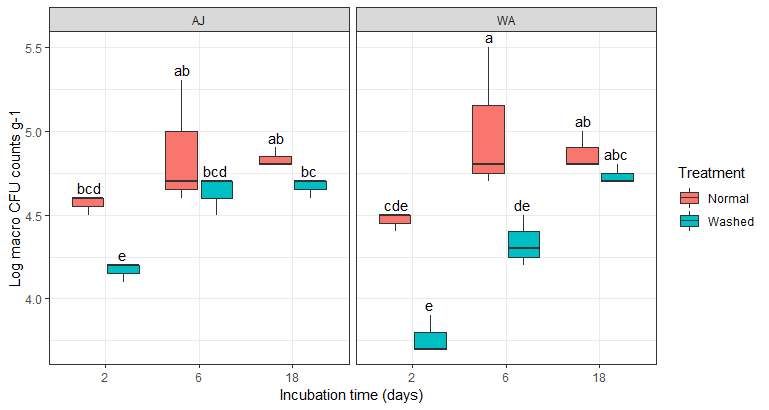


**Fig. S2** Culture-dependent bacterial community analysis of maize phyllosphere: The effect of pre-treatment of inoculum suspension to remove as much as possible of the nutrients traces available in the inocula. Log CFU counts developed on the tested culture media overtime.; statistically significant differences are designated by different letters based on Tukey’s Honestly Significant Differences (HSD, *P* ≤ 0.05, n = 3).

**Table S1** Identification of sunflower rhizobacteria isolates**^*^** based on 16S rRNA gene sequences and closest hits obtained from the EZBioCloud database, and their corresponding isolation source.

| **Top-hit taxon (Isolates Codes ^*^)** | **Similarity (%)** | **Isolation Media** | **Environment/Host** |
| --- | --- | --- | --- |
| - *Agreia* sp. (VKM Ac-1783) - F19 | 99.61 | Trypticase Soy Agar (TSA) | Gall of *Elymus repens* induced by Nematode [8] |
| - *Bacillus aryabhattai* - W16 | 99.65 | Nutrient agar | Air, upper atmosphere [9] |
| - *Curtobacterium flaccumfaciens* - W22, W15, W13, F9, F6, F17, and F11 | ≥ 99.57 | Congo-red Sucrose Agar | Beans stem [10] |
| - *Curtobacterium herbarum* - W8, W6, W5, W3, W24, W10, F4, F14, F13, F10, and F12 | ≥ 99.36 | Nutrient agar II | Mulch of sward [11] |
| - *Herbiconiux flava* - F20 | 98.61 | Corynebacterium agar | Phyllosphere of *Carex* sp. [12] |
| - *Methylobacterium mesophilicum* - W21 | 99.48 | Glucose Yeast Extract Agar | Epiphyllosphere of *Lolium perenne* [13] |
| - *Nocardioides endophyticus* - W18 and W17 | ≥ 99.14 | Reasoner’s 2A agar (R2A) | Endorhizosphere of *Artemisia princeps* [14] |
| - *Nocardioides zeicaulis* - F2 | 98.96 | Trypticase Soy Agar (TSA) | Maize stem [15] |
| - *Plantibacter flavus* - W7 | 99.64 | Nutrient agar | Mulch of sward [11] |
| - *Rhodococcus cercidiphylli* - F8 | 99.77 | Tap-water Yeast Extract Agar [5] | Endophyllosphere of *Cercidiphyllum japonicum* [16] |
| - *Rhodococcus enclensis* - W4 | 99.26 | Marine agar | Marine sediment [17] |

^*^ W, codes of isolates recovered from water agar plates; F, codes of isolates recovered from sunflower filtered juice (FJ) agar plates.

**References**

1 Reasoner DJ, Geldreich E. A new medium for the enumeration and subculture of bacteria from potable water. *Appl Environ Microbiol* 1985; 49: 1-7.

2 Hegazi N, Hamza M, Osman A, Ali S, Sedik M, Fayez M. Modified combined carbon N-deficient medium for isolation, enumeration and biomass production of diazotrophs. *Nitrogen fixation with non-legumes*. Springer, 1998, pp 247-253.

3 Dong Z, Canny MJ, McCully ME, Roboredo MR, Cabadilla CF, Ortega E *et al*. A nitrogen-fixing endophyte of sugarcane stems (a new role for the apoplast). *Plant Physiology* 1994; 105: 1139-1147.

4 Sarhan MS, Mourad EF, Hamza MA, Youssef HH, Scherwinski AC, El-Tahan M *et al*. Plant powder teabags: a novel and practical approach to resolve culturability and diversity of rhizobacteria. *Physiol Plant* 2016; 157: 403-413.

5 Muyzer G, de Waal EC, Uitterlinden AG. Profiling of complex microbial populations by denaturing gradient gel electrophoresis analysis of polymerase chain reaction-amplified genes coding for 16S rRNA. *Appl Environ Microbiol* 1993; 59: 695-700.

6 Mühling M, Woolven-Allen J, Murrell JC, Joint I. Improved group-specific PCR primers for denaturing gradient gel electrophoresis analysis of the genetic diversity of complex microbial communities. *The ISME journal* 2008; 2: 379.

7 Kumar S, Stecher G, Li M, Knyaz C, Tamura K. MEGA X: Molecular Evolutionary Genetics Analysis across Computing Platforms. *Mol Biol Evol* 2018; 35: 1547-1549.

8 Evtushenko L, Dorofeeva L, Dobrovolskaya T, Subbotin S. Coryneform bacteria from plant galls induced by nematodes of the subfamily Anguininae. *Russ J Nematol* 1994; 2: 99-104.

9 Shivaji S, Chaturvedi P, Begum Z, Pindi PK, Manorama R, Padmanaban DA *et al*. Janibacter hoylei sp. nov., Bacillus isronensis sp. nov. and Bacillus aryabhattai sp. nov., isolated from cryotubes used for collecting air from the upper atmosphere. *International journal of systematic and evolutionary microbiology* 2009; 59: 2977-2986.

10 Hedges F. A bacterial wilt of the bean caused by Bacterium flaccumfaciens nov. sp. *Science (Washington)* 1922; 55: 433-434.

11 Behrendt U, Ulrich A, Schumann P, Naumann D, Suzuki K-i. Diversity of grass-associated Microbacteriaceae isolated from the phyllosphere and litter layer after mulching the sward; polyphasic characterization of Subtercola pratensis sp. nov., Curtobacterium herbarum sp. nov. and Plantibacter flavus gen. nov., sp. nov. *International journal of systematic and evolutionary microbiology* 2002; 52: 1441-1454.

12 Hamada M, Komukai C, Tamura T, Evtushenko LI, Vinokurova NG, Suzuki K-i. Description of Herbiconiux flava sp. nov. and emended description of the genus Herbiconiux. *International journal of systematic and evolutionary microbiology* 2012; 62: 795-799.

13 Andreote FD, Lacava PT, Gai CS, Araújo WL, Maccheroni J, Walter, van Overbeek LS *et al*. Model plants for studying the interaction between Methylobacterium mesophilicum and Xylella fastidiosa. *Canadian journal of microbiology* 2006; 52: 419-426.

14 Kim YR, Kim T-S, Han J-H, Joung Y, Park J, Kim SB. Allohumibacter endophyticus gen. nov., sp. nov., isolated from the root of wild Artemisia princeps (mugwort). *International journal of systematic and evolutionary microbiology* 2016; 66: 1823-1827.

15 Kämpfer P, Glaeser SP, McInroy JA, Busse H-J. Nocardioides zeicaulis sp. nov., an endophyte actinobacterium of maize. *International journal of systematic and evolutionary microbiology* 2016; 66: 1869-1874.

16 Li J, Zhao G-Z, Chen H-H, Qin S, Xu L-H, Jiang C-L *et al*. Rhodococcus cercidiphylli sp. nov., a new endophytic actinobacterium isolated from a Cercidiphyllum japonicum leaf. *Systematic and applied microbiology* 2008; 31: 108-113.

17 Dastager SG, Mawlankar R, Tang S-K, Krishnamurthi S, Ramana VV, Joseph N *et al*. Rhodococcus enclensis sp. nov., a novel member of the genus Rhodococcus. *International journal of systematic and evolutionary microbiology* 2014; 64: 2693-2697.
